# ^188^Re-Liposome Can Induce Mitochondrial Autophagy and Reverse Drug Resistance for Ovarian Cancer: From Bench Evidence to Preliminary Clinical Proof-of-Concept

**DOI:** 10.3390/ijms18050903

**Published:** 2017-04-25

**Authors:** Chia-Ming Chang, Keng-Li Lan, Wen-Sheng Huang, Yi-Jang Lee, Te-Wei Lee, Chih-Hsien Chang, Chi-Mu Chuang

**Affiliations:** 1 School of Medicine, National Yang-Ming University, Taipei 112, Taiwan; cm_chang@vghtpe.gov.tw; 2 Department of Obstetrics and Gynecology, Taipei Veterans General Hospital, Taipei 112, Taiwan; 3 Department of Biomedical Imaging and Radiological Sciences, National Yang-Ming University, Taipei 112, Taiwan; kengli@ym.edu.tw (K.-L.L.); yjlee2@ym.edu.tw (Y.-J.L.); chchang@iner.gov.tw (C.-H.C.); 4 Institute of Traditional Medicine, School of Medicine, National Yang-Ming University, Taipei 112, Taiwan; 5 Department of Oncology, Taipei Veterans General Hospital, Taipei 112, Taiwan; 6 Institute of Oral Biology, National Yang-Ming University, Taipei 112, Taiwan; 7 Departments of Nuclear Medicine Radiology, Taipei Veterans General Hospital, Taipei 112, Taiwan; wshuang2@vghtpe.gov.tw; 8 Biophotonics and Molecular Imaging Research Center (BMIRC), National Yang-Ming University, Taipei 112, Taiwan; 9 Isotope Application Division, Institute of Nuclear Energy Research, Taoyuan 325, Taiwan; twlee@iner.gov.tw

**Keywords:** ovarian cancer, cancer stem cells, autophagy, mitophagy

## Abstract

Despite standard treatment, about 70% of ovarian cancer will recur. Cancer stem cells (CSCs) have been implicated in the drug-resistance mechanism. Several drug resistance mechanisms have been proposed, and among these, autophagy plays a crucial role for the maintenance and tumorigenicity of CSCs. Compared to their differentiated counterparts, CSCs have been demonstrated to display a significantly higher level of autophagy flux. Moreover, mitophagy, a specific type of autophagy that selectively degrades excessive or damaged mitochondria, is shown to contribute to cancer progression and recurrence in several types of tumors. Nanomedicine has been shown to tackle the CSCs problem by overcoming drug resistance. In this work, we developed a nanomedicine, ^188^Re-liposome, which was demonstrated to target autophagy and mitophagy in the tumor microenvironment. Of note, the inhibition of autophagy and mitophagy could lead to significant tumor inhibition in two xenograft animal models. Lastly, we presented two cases of recurrent ovarian cancer, both in drug resistance status that received a level I dose from a phase I clinical trial. Both cases developing drug resistance showed drug sensitivity to ^188^Re-liposome. These results suggest that inhibition of autophagy and mitophagy by a nanomedicine may be a novel strategy to overcome drug resistance in ovarian cancer.

## 1. Introduction

In spite of extensive research on epithelial ovarian cancer, this intractable disease remains the leading cause of deaths among patients who were diagnosed with gynecological cancers. Under current standard treatment, about 70% of epithelial ovarian cancer in advanced stage will recur [1,2]. The existence of cancer stem cells has been implicated in the recurrence of epithelial ovarian cancer. Current chemotherapies can kill the bulk of cancer cells, but often are not able to eliminate the critical cancer stem cells (CSCs), which are protected by specific resistance mechanisms.

Current evidence suggests that the resistance mechanisms of cancer stem cells may include deregulated autophagy, enhanced DNA damage response, resistance to reactive oxygen species and altered metabolism status [3]. Of note, most of the above-mentioned pathways are mediated by redox misbalance and involvement of mitochondria-mediated antioxidant capacity [4].

Among the aforementioned resistance mechanisms, autophagy plays a crucial role for the maintenance and tumorigenicity of CSCs [5,6]. Compared to their more differentiated counterparts, CSCs have been demonstrated to display a significantly higher level of autophagy flux. Moreover, mitophagy, a specific type of autophagy that selectively degrades excessive or damaged mitochondria, is shown to contribute to cancer progression and recurrence in various types of tumors [7,8]. It has recently been found that the inhibition of autophagy/mitophagy enhances chemosensitivity or overcomes drug resistance in cancer cells [9,10].

Nanotechnology has shown significant promise in the development of drugs and drug delivery systems that can overcome the obstacles of drug resistance and address urgent needs to improve diagnostic efficacy and therapy of various diseases [11,12]. In the past two decades, examples of nanotechnology-based approaches to tackle the CSCs problem have been accumulating. Some groups have successfully demonstrated using nanomedicines to target the CSCs to eliminate the tumor and prevent recurrence [13,14]. Furthermore, Dakwar had shown that intraperitoneal therapies would be able to retain anti-cancer drugs as much as possible in the peritoneal cavity, to achieve maximal tumor exposure to the drugs [15].

In the past ten years, our researchers have developed a nanomedicine, ^188^Re-liposome, which was designed to be composed of radioisotope ^188^Re conjugated to an *N*,*N*-bis(2-mercaptoethyl)-*N*_9_,*N*_9_-diethylethylenediamine (BMEDA) chelator and embedded in liposomes, which have been proven to be effective for targeting metastatic lung and colorectal cancers in animal models [16,17,18]. Further, we also have demonstrated that ^188^Re-liposome is effective in the suppression of stemness markers’ expression and switches from glycolysis to the oxidative phosphorylation (OXPHOS) in an animal model of ovarian cancer [19]. 

In the current work, we aimed to explore the capability of inhibiting CSCs by ^188^Re-liposome. Free drug (^188^Re-BMEDA) and nanomedicine (^188^Re-liposome), by different routes of delivery (systemic or intraperitoneal), were tested. We show that ^188^Re-liposome could effectively suppress the function of CSCs by targeting autophagy/mitophagy. Further, we show the anti-tumor effect of in vivo animal model. Lastly, we provide preliminary clinical data of two cases of ovarian cancer, reporting the capability of ^188^Re-liposome to reverse from drug resistance status to drug sensitive status and successfully extend the expected survivorship. 

## 2. Results

### 2.1. Manufacture and Administration of ^188^Re-Liposome

**Figure 1:**
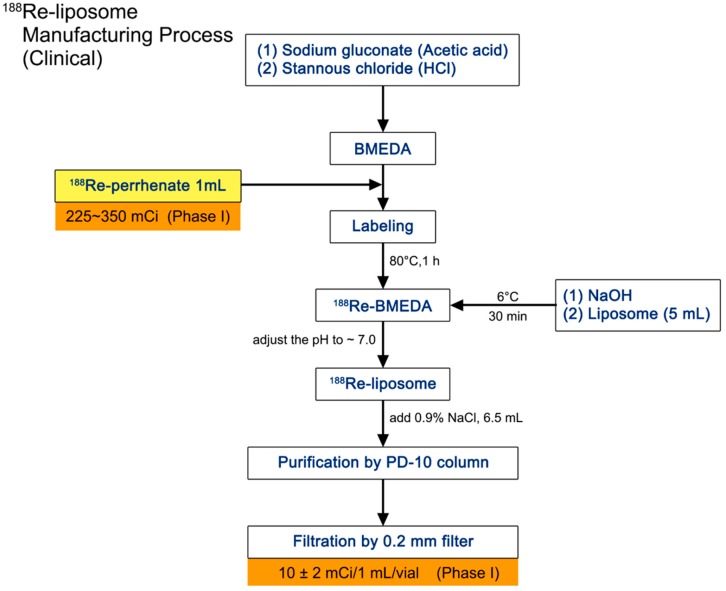
Schematic diagram of preparing ^188^Re-liposome. The manufacturing process includes embedment of liposome and ^188^Re-perrhenate. The final product would be aliquoted into vials in 10 ± 2 mCi per 1 mL in the phase I trial. BMEDA, *N*,*N*-bis(2-mercaptoethyl)-*N*_9_,*N*_9_-diethylethylenediamine.

The production process of ^188^Re-liposome is detailed in the flow chart and was performed in a good manufacturing practices (GMP)-qualified laboratory (Figure 1). The radioactivity of each batch of ^188^Re-liposome was examined and measured each time before injection. The quality control was proceeded in each production to meet the following criteria. The pH should be between 6 and 8, and radiochemical purity needed to be more than 90% by the TLC scanner. Radioactivity was 14 ± 3 mCi and showed a 155-keV peak at the γ spectrum. In addition, the purity of the radionuclide could not be lower than 99.9%. The phospholipid concentration would be 3–6 μmol/mL. The characteristics of liposome were also defined to have a particle size of 80–100 nm, z-potential between −3 and 2 mV and BMEDA not exceeding 175 μg/mL. Last but not least, endotoxin concentration should be equal to or lower than 175 EU/mL. 

For the first clinical case, the standard sample was 94.8 μCi and 90.2 μCi per 10 μL in stages I and II separately, and the exact radioactivity injecting to the patient was 14 mCi (stage I) and 19.32 mCi (stage II). For the second clinical case, the standard sample was 74 μCi and 84.3 μCi per 10 μL in stages I and II separately, and the actual administered dose was 14 mCi (stage I) and 24.65 mCi (stage II). 

### 2.2. Intraperitoneal Delivery of ^188^Re-Liposome Accumulates the Most in the Xenograft Mice Model

**Figure 2:**
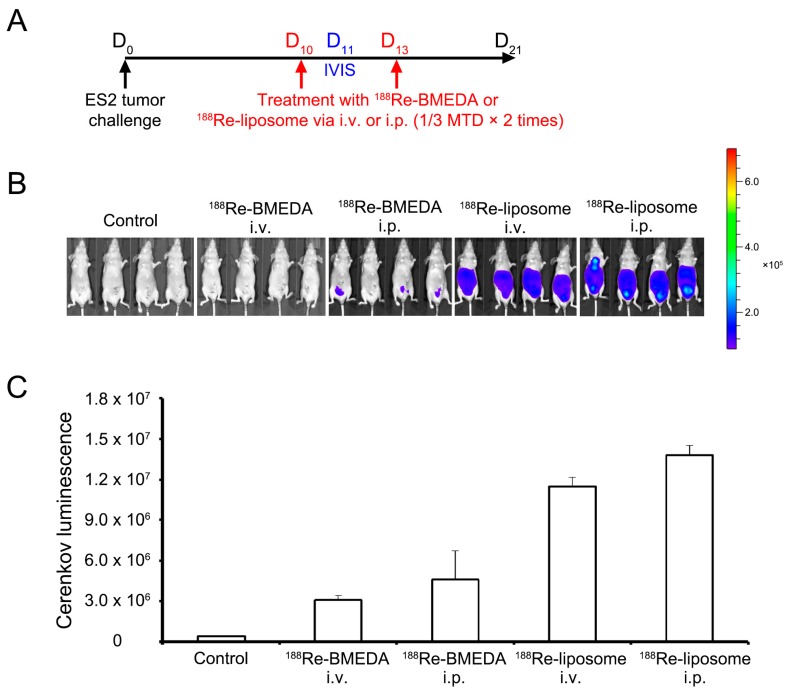
In vivo ^188^Re-liposome retention radioactivity in the xenograft mice model. (**A**) Schematic diagram of the treatment regime showing the timing schedule; (**B**) Luminescence images of representative mice receiving various treatment regimens. The color represented the intensity of ^188^Re radioactivity from minimum 9 × 10^4^ (blue) to maximum 7 × 10^5^ (red); (**C**) Sum of quantitative luminescent photons of in vivo imaging system (IVIS) images in (**B**). Data are presented as the mean ± SD with quadruplicates. BMEDA, *N*,*N*-Bis(2-mercaptoethyl)-*N*′,*N*′-diethylethylenediamine; MTD, maximal tolerated dose; IVIS, in vivo imaging system; i.v., intravenous; i.p., intraperitoneal.

To evaluate which delivery route and drug form provides the longest duration of ^188^Re in the mice model, we injected the mice with nearly 1/3 maximal tolerated dose (MTD) or 360 μCi at Day 10, and took the IVIS image the next day (Figure 2A). This image clearly showed that the ^188^Re-liposome formed in either route stayed in the peritoneal cavity more stable than the BMEDA forms (Figure 2B). Furthermore, the intraperitoneal delivery route treated regionally was able to accumulate higher radioactivity than the intravenous route in either the liposome- or BMEDA-form of ^188^Re. Among the treatments, intraperitoneal delivery of ^188^Re-liposome was the most effective therapeutic strategy to retain drug in mice up to 1.3 × 10^7^ Cerenkov luminescence, which indicated the intensity of the ^188^Re radionuclide (Figure 2C).

### 2.3. In Vivo Analysis of ^188^Re-Liposome on Targeting Autophagy and Mitophagy

**Figure 3:**
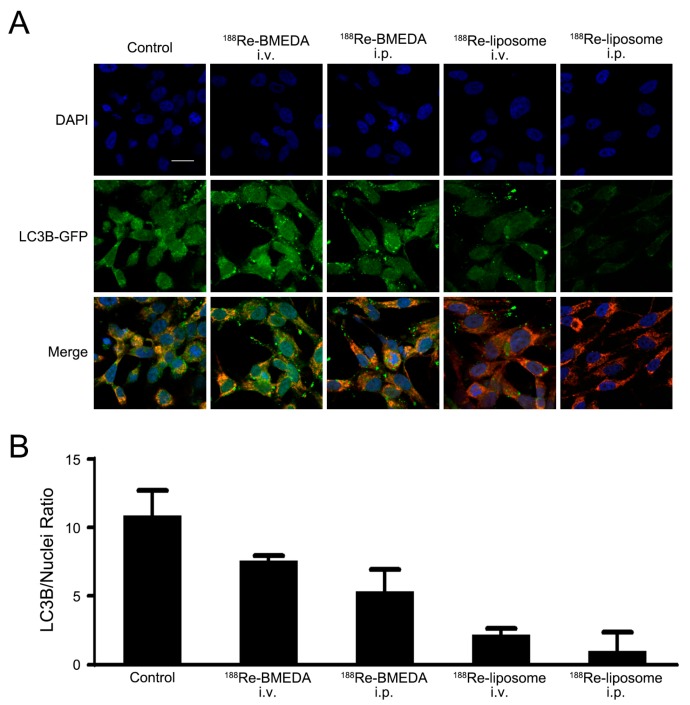
Intraperitoneal delivery of ^188^Re-liposome revealed a critical role in altering LC3B. (**A**) Images of live cells from primary cultures were taken by confocal microscopy. Treatment with ^188^Re-liposome significantly suppressed the expression of LC3B in xenograft mice models. Representative data are shown, (**B**) each bar depicting the ratio of LC3B/nuclei from (**A**). Data are presented as the mean ± SD with at least triplicates. DAPI, 4′,6-diamidino-2-phenylindole; GFP, Green fluorescent protein; LC3B, microtubule-associated protein 1 light chain 3B. Scale bar represents 20 μm.

The localization of microtubule-associated protein 1 light chain 3B (LC3B) was mostly present in the surface mitochondria, which was shown by bound LC3B-GFP monoclonal antibody compared with 4′,6-diamidino-2-phenylindole (DAPI)-dyed nuclei (Figure 3A). The descending pattern of LC3B/nuclei under each treatment was closely related to the radioactivity pattern (Figure 3B), and this may indicate the direct or indirect relationship between ^188^Re radionuclide and autophagy.

**Figure 4:**
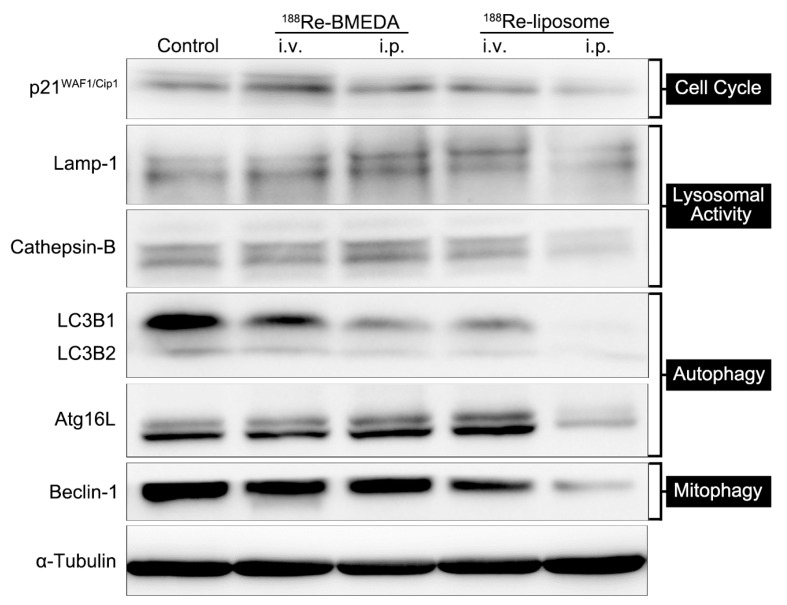
Intraperitoneal delivery of ^188^Re-liposome downregulates proteins of the cell cycle, lysosomal activity, selective and non-selective autophagy. The expression of related proteins is shown by Western blotting analysis and presented subsidence of p21^WAF/Cip1^, Lamp-1, cathepsin-B, LC3B, Atg16L and Becline-1 upon ^188^Re-liposome i.p. treatment. Representative data are shown from triplicate experiments. LC3B, microtubule-associated protein 1 light chain 3B; Atg16L, autophagy related 16-like gene.

In addition, intraperitoneal delivery of ^188^Re-liposome reduced lysosomal proteins, including Lamp-1 and cathepsin-B and p21^WAF/Cip1^ as the cell cycle regulator (Figure 4). Suppressed lysosomal activity solidified the decline of mitophagy and autophagy. For an explanation of diminished p21^WAF/Cip1^ expression, despite its well-known role in cell cycle arrest, there was research suggesting that p21 was able to promote proliferative activity and oncogenicity [20].

We found that regional delivery of sustained release ^188^Re-liposome via intraperitoneal injection significantly reduced and downregulated non-selective autophagy and selective autophagy, as demonstrated by the reduction of LC3B, Atg16L and Beclin-1 (Figure 4) after the administration of ^188^Re-liposome.

### 2.4. In Vivo Intraperitoneal Delivery of ^188^Re-Liposome Is Able to Inhibit Tumor Growth of Two Different Cell Lines in Mice Models

**Figure 5:**
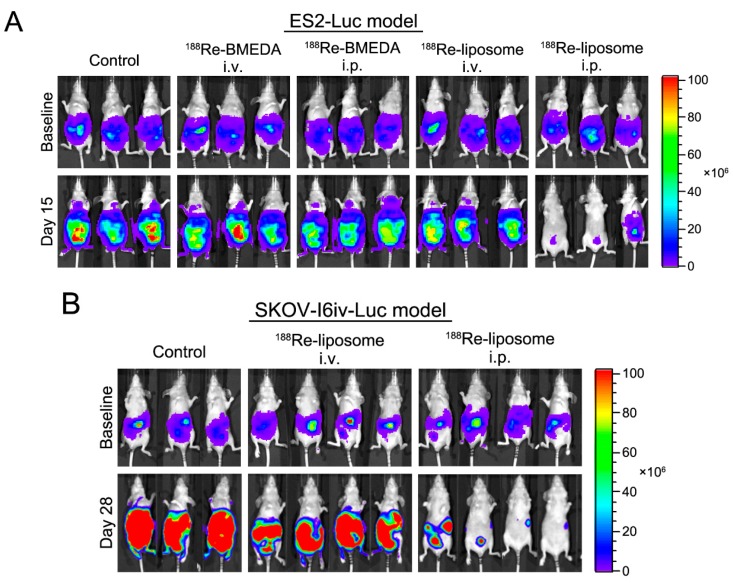
Intraperitoneal delivery of ^188^Re-liposome offers significant tumor killing effects. Tumors originating from ES2-luc (**A**) and SKOV-I6-luc (**B**) cells were detected at Day 15 and 28 in the abdominal region of xenograft mice models. The In Vivo Bioluminescence Imaging System (IVIS) was applied to measure the intensity of tumor cells. Representative data are shown from triplicate (**A**,**B**) or quadruplicate (**B**) experiments.

From the analysis of IVIS images, tumors (ES-2 cells) were poorly controlled under the treatments of the free form of ^188^Re-BMEDA in either routes or ^188^Re-liposome in intravenous routes (Figure 5A). In contrast, tumors of both cell lines (ES-2 and SKOV-I6iv-luc) were remarkably reduced by ^188^Re-liposome via the intraperitoneal pathway (Figure 5A,B). Taking this with the fact of ^188^Re accumulation (Figure 1B) into consideration, the in vivo data strongly suggested that longer retention time in the peritoneal cavity of ^188^Re-liposome was able to exert a significant anti-tumor effect against ovarian cancer.

### 2.5. Clinical Case Presentation

**Figure 6:**
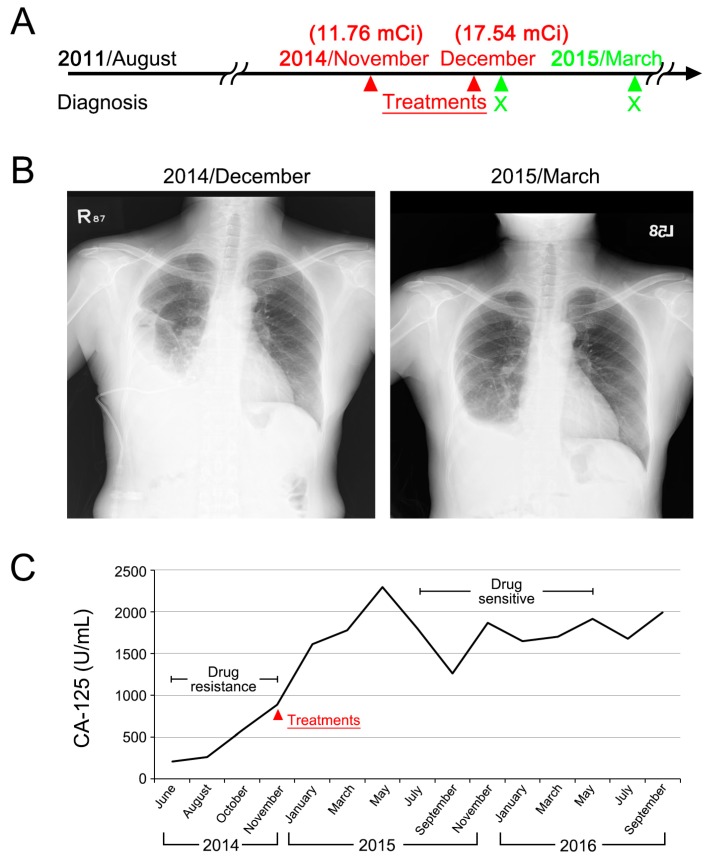
Case 1 presentation of a ^188^Re-liposome phase I trial. (**A**) Clinical treatment scheme; (**B**) chest film before and after ^188^Re-liposome treatment; (**C**) monitoring of serum CA-125.

The first case, aged 58, was first diagnosed stage IIIC serous adenocarcinoma of ovary in August 2011. She had received multiple regimens of chemotherapy after the first recurrence. In November 2014, she presented with extensive peritoneal spreading combined with right pleural effusion. Gradual elevation of Cancer Antigen 125 (CA-125) was noted despite chemotherapy. She received ^188^Re-liposome in November (11.76 mCi) and in December (17.54 mCi). In May 2015, about four months after the last treatment, CA-125 started to decline. In addition, chest film followed-up in March 2015 showed partial resolution of right pleural effusion (Figure 6). As of January 2017, this case was still in good performance status.

**Figure 7:**
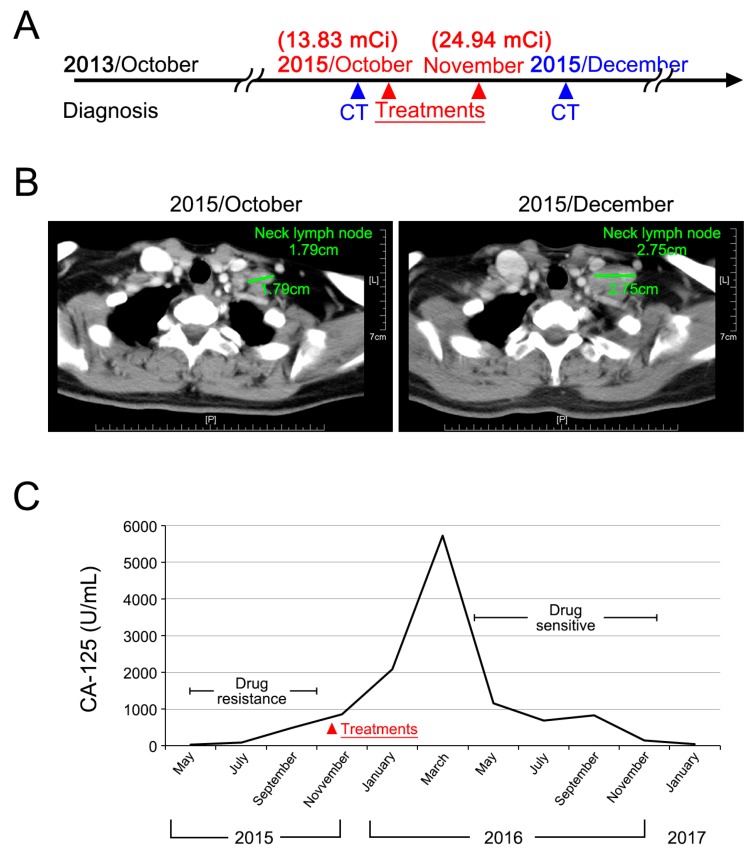
Case 2 presentation of a ^188^Re-liposome phase I trial. (**A**) Clinical treatment scheme; (**B**) CAT scan imaging before and after ^188^-Re-liposome treatment. Enlargement of supraclavicular lymph nodes is presumed to be due to the acute inflammatory reaction of lymph nodes; (**C**) Monitoring of serum CA-125.

The second case, aged 51, was first diagnosed stage IIIC endometrioid adenocarcinoma of ovary in October 2013. She had received multiple regimens of chemotherapy after the first recurrence. In October 2015, she presented with extensive peritoneal seeding, and a left supraclavicular lymph node measured 1.79 cm. She received ^188^Re-liposome in October (13.83 mCi) and in November (24.94 mCi). In March 2016, CA-125 started to decline. Although the supraclavicular lymph node showed slight enlargement after treatment, this phenomenon was considered to be an acute inflammatory reaction to treatment rather than tumor progression (Figure 7). As of January 2017, this case was still in good performance status.

Although the two cases presented here cannot lead to the conclusion of ^188^Re-liposome being effective or not, however, the extended after treatment life of the two cases is more than expected. In the usual condition, the expected median survival for extensive peritoneal spreading of ovarian cancer is less than one year due to the complication of intestinal bowel obstruction.

## 3. Discussion

In this work, our data reveal that ^188^Re-liposome can significantly inhibit the function of CSCs by suppressing autophagy/mitophagy in ovarian cancer cells. Though the exact determination of CSCs was not conducted in the experiment, those cells surviving under radiotherapy would be strongly resistant to treatment, and if we recall that CSCs always hold out until the last, it is not surprising that most of the remaining cells would be considered as CSCs. This phenomenon can be translated into tumor suppression in an animal model. Lastly, two ovarian cancer patients demonstrated reversal from drug resistance status to drug sensitive status after injection of ^188^Re-liposome. From the clinical perspective, ^188^Re-liposome paves the way for investigating drug resistance by using a strategy of inhibiting the function of CSCs.

One of the major concerns in current cancer research is drug resistance to chemotherapy and targeted therapies. A various range of molecular mechanisms contributes to drug resistance including autophagy, increased rates of drug efflux, metabolic alternation and mutation of drug targets [21,22,23].

Among the aforementioned drug resistance mechanisms, autophagy essentially plays an important role in cellular homeostasis. This normal physiological mechanism, which responds to defective or damaged organelles, activates fusion with lysosomes to digest mitochondria [24], endoplasmic reticulum [25] and peroxisomes [26]. A number of investigations suggest that autophagy induction can promote resistance to cell death within tumor cells and play a critical role in resistance to chemotherapy in cancer treatment [27]. Besides traditional cancer hallmarks, now the regulation of autophagy is recognized as a new hallmark of cancer cells. Autophagy is able to facilitate survival of cancer cells through conquering metabolic stress and chemotherapeutic cytotoxicity. For example, pancreatic cancer cells showed elevated autophagy in cases when treated with chemotherapy [28,29]. In addition, chloroquine, hydroxychloroquine and other autophagy inhibitors with chemotherapy and radiotherapy have been presented to perform a synergistic effect [30].

Another type of selective autophagy, called mitophagy, serves for the removal of dysfunctional mitochondria from the cells and is often controlled by a moderate level of reactive oxygen species (ROS) [31,32]. During mitophagy, dysfunctional mitochondria are engulfed by a double-layered membrane (phagophore) and form so-called autophagosome followed by degradation [33]. Recently, the concept of an autophagic tumor-stroma model of cancer cell metabolism that is relevant to mitophagy has been advocated. According to this model, tumor-released ROS induce oxidative stress in adjacent tumor-associated fibroblasts. These permissive fibroblasts may respond at two levels: first, by producing more ROS, which in turn promote DNA damage and aneuploidy in the malignant cells (i.e., genomic instability), and secondly, these tumor-associated fibroblasts enter into a ‘reverse Warburg effect’, whereby they can undergo autophagy/mitophagy and aerobic glycolysis that eventually leads the tumor microenvironment to be more prone to tumor progression and drug resistance [34].

From a clinical standpoint, some trials have showed the efficacy of autophagy inhibitors. For example, the combination of a mechanistic target of rapamycin (mTOR) inhibitor and an autophagy inhibitor AZD5363 has demonstrated clinical efficacy against solid tumors, while either drug alone did not [35]. A phase II study focusing on the efficacy of sorafenib in patients with refractory lymphoma showed a significant reduction in a certain autophagic biomarker during treatment, even though these patients had higher baseline levels of the marker when they clinically were responsive to sorafenib [36]. According to the ClinicalTrials website, there are two phase II trials currently continuing to appraise the effect of lysomotropic autophagic inhibitor chloroquine alone in patients having breast cancer or ductal carcinoma in situ before surgery. Lastly, when used in combination with anticancer agents, hydroxychloroquine (autophagy inhibitor) improves the efficacy of the standard therapy by inducing autophagy; this evidence justifies the approval by the The Food and Drug Administration (FDA) and its current use in phase I, II and III studies [37].

In the setting of recurrent ovarian cancer, chemotherapy still remains the cornerstone of treatment. Several platinum-based combination chemotherapy regimens dependent on the platinum-free interval are commonly used, despite the fact that most of these regimens have failed to improve the overall survival [38,39,40]. Of note, a recent published phase III trial (HECTOR trial) has demonstrated that combination of topotecan and carboplatin failed to confer superior survival compared to standard regimens [41]. Therefore, it is imperative to develop a more effective therapeutic strategy to treat this intractable disease.

Nanotechnology has the potential to overcome drug resistance induced by chemotherapy because of the unique properties of nanoparticles [42]. By taking advantage of the so-called enhanced and permeability (EPR) effect, nanoparticles can be constructed at a certain size (100–300 nm) for effective biodistribution and accumulation in the tumor microenvironment [43]. For example, a novel liposome encapsulated bile acid-cisplatin complex, called Bamet-R2, was synthesized with the aim of overcoming cisplatin resistance [44]. Further, a liposomal cisplatin derivative, nano-diamminedichloroplatinum (NDDP) was not cross-resistant with cisplatin in either in vitro or in vivo systems and was shown to be more active than free cisplatin against tumor metastasis [45].

To date, autophagy inhibition to control CSCs by the nanomedicine-based strategy has rarely been reported [46]. Compared with single treatment, the combined delivery systems of nanoparticle-encapsulated chloroquine (autophagy inhibitor) and nanoparticle-encapsulated doxorubicin or docetaxel showed the most effective and persistent tumor growth inhibitory effect by eliminating bulk tumor cells, as well as CSCs in an MDA-MB-231 orthotopic breast cancer murine model. In the current work, we also demonstrated that a nanomedicine, ^188^Re-liposome, revealed significant autophagy/mitophagy inhibition compared to the control group or ^188^Re-BMEDA, a free ^188^Re drug. Nonetheless, certain markers (p21, LAMP-1 and cathepsin-B) increased in the ^188^Re-BMEDA group. We proposed that cancer stem cell signals were enhanced for this group, as several lines of evidence have proven increased stemness signal after chemotherapy or radiation treatment [47,48].

## 4. Materials and Methods

### 4.1. Cell Lines and Animal Models

The human ovarian clear cell ES2 and carcinoma SKOV-3 cell line were purchased from the American Type Culture Collection (ATCC), Manassas, VA. These cell lines were cultured in McCoy’s 5A (Corning Mediatech, Inc., Manassas, VA, USA) with 10% fetal bovine serum (FBS) (HyClone, Logan, UT, USA). The ES2-luc cell that stably expresses the firefly luciferase gene was generously provided by the Tzyy-Choou Wu lab at Johns Hopkins University. ES2-luc was cultured in complete Dulbecco’s Modified Eagle Medium (DMEM), furnished with 1% sodium pyruvate; 1% non-essential amino acids; 1% antibiotics with penicillin and streptomycin sulfate; and 10% FBS. The SKOV-I6iv-luc cell line is a highly invasive cell line, which was kindly provided by Dr. Lu-Hai Wang (Institute of Molecular and Genomic Medicine, National Health Research Institute, Miaoli County, Taiwan). SKOV-I6iv-luc was maintained in DMEM culture medium contained 10% FBS, 1% penicillin and streptomycin sulfate and G418 (800 μg/mL).

To evaluate the therapeutic efficacy, 1 × 10^6^ ES2-luc stable cell lines with luciferase activity were inoculated into 5-week-old BALB/c nude mice with intraperitoneal injection at Day 0. The ES2-luc tumor-bearing mice (6 mice per group) were intravenously or intraperitoneally injected twice with 24.67 MBq/100 μL (2/3 MTD) ^188^Re-BMEDA or ^188^Re-liposome respectively at Days 5 and 12. The therapeutic efficacy was assessed by the bioluminescent signal acquired from the In Vivo Imaging System (IVIS 50, Perkin Elmer Inc., Waltham, MA, USA) at Day 4 (baseline) and Day 15. Another ovarian cancer ascites model, 1 × 10^6^ SKOV-I6iv-luc cells were implemented via intraperitoneal injection, followed by twice ^188^Re-liposome treatment at Day 5 and 12 by the intravenous or intraperitoneal route. The bioluminescence images were captured on Day 4 (baseline) and Day 28.

To acquire the different formulation of ^188^Re-treated (^188^Re-liposome or ^188^Re-BMEDA) ovarian cancer cells for the sequel analysis, ES2 cells were orthotopically inoculated into the surface region of ovary in 5-week-old BALB/c nude mice. Then, 1 × 10^7^ ES2 cells were diluted in 1 mL Hank’s balanced salt solution (HBSS). The mice were anesthetized by a mixture of Zoletil and Rompun solution (1:1 ratio, 1 mL/kg intraperitoneally) and placed vertically. ES2 cells were inoculated under the ovarian bursa (using a 30-gauge needle syringe) after a small abdomen incision through surgical operation. The dosing schedule is shown in Figure 2A. At Day 10 and 13 post-tumor inoculation, the tumor-bearing mice received 1/3 MTD of ^188^Re-BMEDA or^188^Re-liposome by either intravenous or intraperitoneal injection. The mice were sacrificed at Day 21, and tumors were then extracted directly for downward experiments. Based on the high energy β particles emitted from ^188^Re, Cerenkov luminescent imaging (CLI) can be utilized to monitor the accumulation of ^188^Re-liposome by the In Vivo Imaging System (IVIS 50, Perkin Elmer Inc., Waltham, MA, USA), which was more sensitive than detecting the radioactive signals [49]. The CLI in the tumor-bearing mouse after 24 h of ^188^Re-liposome or ^188^Re-BMEDA injection was performed subsequently by using IVIS.

The experimental animals were purchased from BioLASCO Taiwan Co., Ltd. (Taipei, Taiwan), and housed in the animal facility of Taipei Veterans General Hospital, Taipei, Taiwan. These animals were applied in compliance under the institutional animal healthcare regulations, and all animal experimental procedures were approved by the Institutional Animal Care and Use Committee (IACUC 2015-122 and 2016-163, permission date: 3 August 2015) of Taipei Veterans General Hospital. 

### 4.2. Preparation of ^188^Re-BMEDA and ^188^Re-Liposome 

The procedure of preparing the radioactive material-embedded liposome is exhibited in Figure 1. Briefly, ^188^Re was milked from an ^188^W/^188^Re generator (Institut National des Radioelements, Fleurus, Belgium). The eluted sodium perrhenate (Na^188^ReO_4_) was inoculated with BMEDA (ABX GmbH) in a water bath at 80 °C. The PEGylated liposome (phospholipids (13.16 mmol/mL)) (NanoX; Taiwan Liposome Co., Ltd., Taipei, Taiwan) was mixed with the solution containing ^188^Re-BMEDA for another 30 min incubation in a 60 °C water bath. The PD-10 chromatographic column (GE Healthcare, Little Chalfont, UK) was used to isolate embedded liposome from free ^188^Re-BMEDA.

### 4.3. Western Blot Assay

The whole-cell extracts were prepared by lysis buffer supplement with various protein inhibitors. Equal protein aliquots of each lysate were subjected to SDS-PAGE (6–12%), transferred onto polyvinylidene difluoride (PVDF) membrane (Millipore, Bedford, MA, USA). After blocking with blocking solution for 1 h, the PVDF membranes were probed with specific antibodies overnight at 4 °C, followed by incubation with corresponding secondary antibody for 1 h at room temperature and subsequently detected by chemiluminescence. Protein concentration was determined by the Micro BCA Protein Assay Kit (Pierce Biotechnology, Waltham, MA, USA).

Anti-α-tubulin (1:1000, ab4074) was from Abcam (Abcam, Cambridge, MA, USA), and anti-p21^Waf1/Cip1^ (1:1000, 2947S), Lamp1 (1:1000, 9091S), cathepsin (1:1000, 3373S), LC3B (1:1000, 3868S) Atg16L (1:1000, 8089S) and Beclin-1 (1:1000, 3495S) were from Cell Signaling (Danvers, MA, USA).

### 4.4. Autophagy Detect and Mitochondrial Morphology Analysis

To detect the autophagosome, the treated ES2 grown on glass coverslips were infected with BacMam LC3B-GFP vectors (Invitrogen, Carlsbad, CA, USA) by GenJet In Vitro DNA transfection reagent (SignaGen Laboratories, Rockville, MD, USA) and incubated for 24 h at 37 °C. The LC3B-GFP puncta were captured by an Olympus FV10i (Olympus America Inc., Centre Valley, PA, USA) and calculated by using the MetaMorph^®^ Automation and Image Analysis Software (Molecular Devices, Sunnyvale, CA, USA).

### 4.5. Clinical Case Presentation

Two cases of recurrent ovarian cancer that had undergone several regimens of salvage chemotherapy that were in drug resistance status were reported. The two cases presented here received a level 1 dose of ^188^Re-liposome (0.42 ± 0.04 mCi/kg), which was the starting dose of a phase I ^188^Re-liposome clinical trial (ClinicalTrials.gov Identifier: NCT02271516). The clinical trials were approved by the Institutional Review Board (IRB2013-02-003A, permission date: 3 February 2013), Taipei Veterans General Hospital.

The definition of drug resistance was elevation of serum CA-125 or tumor progression despite chemotherapy. After ^188^Re-liposome treatment, serial CA-125 was monitored for validating drug response.

### 4.6. Statistical Analysis

All data are presented as the mean ± SD. Statistical analysis was performed using Student’s *t*-test for continuous variables. Survival between each indicated group was analyzed by the log-rank test. *p* < 0.05 was considered statistically significant.

## 5. Conclusions

In conclusion, our data reveal that a nanomedicine, ^188^Re-liposome, could effectively show autophagy/mitophagy inhibition to overcome drug resistance of ovarian cancer, as evidenced by the two case reports that the drug resistance status reversed and turned into drug sensitive status. At the writing of this manuscript, two cases are still alive, and the performance scores are 0/1, implying a low toxicity of ^188^Re-liposome. Currently, there are several ongoing early clinical trials exploiting autophagy inhibition to fight cancer [50]. The aim of the presentation of the two clinical cases is not to present the report of phase I trial of ^188^Re-liposome. Instead, we just present the potential of ^188^Re-liposome to reverse drug resistance status. The results of these trials may change the treatment outlook for ovarian cancer. In the future, randomized multi-institutional studies are justified to confirm the valid potential of this novel strategy.
